# Air-Stable Self-Driven UV Photodetectors on Controllable Lead-Free CsCu_2_I_3_ Microwire Arrays

**DOI:** 10.1021/acsami.3c17881

**Published:** 2024-02-21

**Authors:** Zhi-Hong Zhang, Shan-Shan Yan, Yu-Long Chen, Zhen-Dong Lian, Ai Fu, You-Chao Kong, Lin Li, Shi-Chen Su, Kar-Wei Ng, Zhi-Peng Wei, Hong-Chao Liu, Shuang-Peng Wang

**Affiliations:** † State Key Laboratory of High Power Semiconductor Lasers, Changchun University of Science and Technology, Changchun 130022, China; ‡ Institute of Applied Physics and Materials Engineering, University of Macau, Taipa, Macao SAR 999078, China; § Key Laboratory for Photonic and Electronic Bandgap Materials, Ministry of Education, School of Physics and Electronic Engineering, Harbin Normal University, Harbin 150025, China; ∥ School of Semiconductor Science and Technology, South China Normal University, Foshan 528000, China

**Keywords:** CsCu_2_I_3_, perovskite, ultraviolet photodetection, self-driven photodetector, microwire arrays

## Abstract

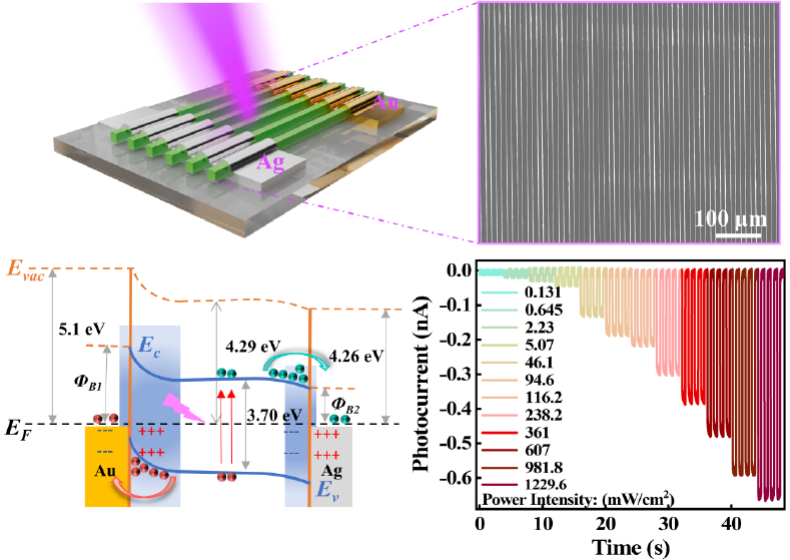

The rapid evolution of the Internet of Things has engendered increased requirements for low-cost, self-powered UV photodetectors. Herein, high-performance self-driven UV photodetectors are fabricated by designing asymmetric metal–semiconductor–metal structures on the high-quality large-area CsCu_2_I_3_ microwire arrays. The asymmetrical depletion region doubles the photocurrent and response speed compared to the symmetric structure device, leading to a high responsivity of 233 mA/W to 355 nm radiation. Notably, at 0 V bias, the asymmetric device produces an open-circuit voltage of 356 mV and drives to a short-circuit current of 372 pA; meanwhile, the switch ratio (*I*_ph_/*I*_dark_) reaches up to 10^3^, indicating its excellent potential for detecting weak light. Furthermore, the device maintains stable responses throughout 10000 UV-light switch cycles, with negligible degradation even after 90-day storage in air. Our work establishes that CsCu_2_I_3_ is a good candidate for self-powered UV detection and thoroughly demonstrates its potential as a passive device.

## Introduction

Over the past few decades, as III-nitrides, metal oxides, SiC, diamond, and other materials^1−4^ have gradually gained public exposure, the field of ultraviolet (UV) detectors has rapidly advanced. This has led to their extensive application in military and civilian fields, including defense warning systems, UV communication, environmental measuring, flame detection, and medical–biological analysis.^5−7^ Notably, the stability of traditional materials is incomparable; however, their preparation demands are high, and the majority of devices shows second-order UV response.^8−10^ While researchers attempt to compensate for this deficiency by designing structures,^11−13^ unfortunately, it also simultaneously poses a significant bottleneck for achieving further breakthroughs in both device figure-of-merits and cost.

Recently, ABX_3_ structured halide perovskite materials^14^ have been widely used as the active material for different functional detectors,^15,16^ owing to their excellent properties such as straightforward solution synthesis method, high light absorption coefficient, balanced bipolar charge mobility, long carrier diffusion length, and flexible structure.^17−19^ Cl-based lead halide perovskites have shown great potential in UV detection.^20,21^ It can achieve a fast response speed in the order of microseconds.^21^ The exceptional performance of the device motivates researchers to address two main challenges associated with the materials: the air stability of the ABX_3_ structure and the toxicity of Pb^2+^ ions at position B. Thanks to the flexibility of the perovskite structure, an all-inorganic lead-free halide material (CsI)_1–*y*_(CuI)_*y*_ formed by completely replacing Pb^2+^ with Cu^+^ was found.^22,23^ This material exhibits a suitable UV absorption broadband and excellent stability against water, oxygen, light, and thermal effects.^24^ It can be stored in air for 80 days to maintain UV detection ability^25,26^ and can work continuously for more than 580 h as yellow phosphors under the condition of relative humidity of 40%,^27^ which will open up a new dimension in UV detection application of perovskite. Several promising results have been demonstrated for CsCu_2_I_3_ UV detection applications, encompassing solar-blind ultraweak light detection,^28^ UV imaging,^29^ and UV polarization detection.^30^ Nevertheless, these devices have relied on an external voltage to power the photogenerated carriers, limiting their independent and sustainable operation, especially as the further development of Internet of Things technology increases the demand for integrable passive devices. Currently, there are no reports available on self-driven UV photodetection using CsCu_2_I_3_, the stable and eco-friendly material.

In this work, a stable self-driven CsCu_2_I_3_ UV photodetector is demonstrated on high-quality CsCu_2_I_3_ microwire arrays (MWAs) with an asymmetric metal–semiconductor–metal (MSM) structure. High-quality CsCu_2_I_3_ MWAs (6 × 3 mm^2^) were achieved via an improved template-restricted assisted crystallization (TRAC) method. Compared to the symmetric (Au–CsCu_2_I_3_–Au) MSM device on the MWAs, the asymmetric Au–CsCu_2_I_3_–Ag structured UV photodetector shows a superior performance. Under the illumination of 355 nm UV, the device exhibits a responsivity of 233 mA/W (at 5 V bias) and a rapid photoresponse speed (τ_rise_/τ_decay_: 2.47 ms/2.46 ms) attributed to the effect of the asymmetric Schottky contact barriers. More importantly, the asymmetric barrier promises the device a typical photovoltaic behavior. The short-circuit current of 372 pA is generated in the device circuit under an open-circuit voltage of 356 mV. Under self-driven mode, the UV responsivity reaches up to 6.5 mA/W, and the UV photocurrent on/off ratio reaches 3 orders of magnitude upon 355 nm irradiation. Furthermore, the self-driven device exhibited minimal performance decay after being stored for 90 days in air and maintained a stable response through tens of thousands of UV on/off cycles. The work presents a potential pathway for the low-cost production of stable self-driven UV photodetectors and serves as a guide for the development of lead-free perovskite detectors.

## Results and Discussion

**Figure 1:**
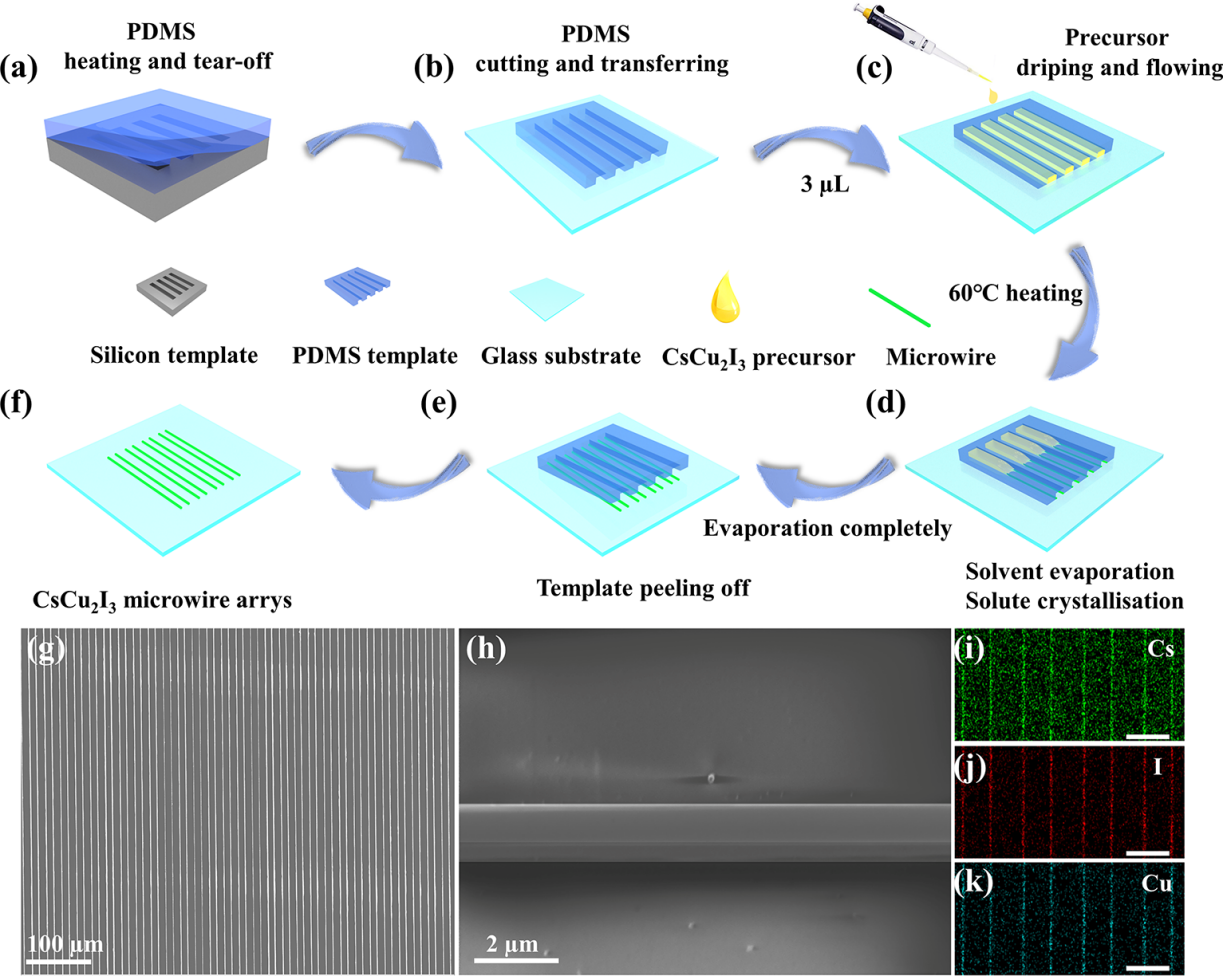
Template-assisted growth and transfer method for high-quality CsCu_2_I_3_ MWAs. (a)–(f) Synthesis scheme of CsCu_2_I_3_ MWAs using the TRAC method: a) PDMS was dropped on the silicon template with preprepared rectangular grooves and heated to solidify. Then the graphical PDMS template was stripped off. b) The PDMS template was transferred to the clean glass substrate by cutting, exposing both ends of the channel. c) A small amount of CsCu_2_I_3_ precursor was dropped at one end of the template, filling the template channel through infiltration. d) Heating caused the solvent volatilization and solute to crystallize along the channel sidewalls. e) After complete evaporation of the solvent, the PDMS template was then gently peeled off. f) Uniform microwire arrays were obtained on substrates. SEM image of (g) the large area CsCu_2_I_3_ MWA and (h) a single CsCu_2_I_3_ microwire on Si substrates. (i–k) EDS elements mapping of the CsCu_2_I_3_ MWAs. Green: cesium; red: iodine; blue: copper (scale bars,10 μm).

The CsCu_2_I_3_ MWAs were successfully fabricated by a simple and efficient template restriction-assisted crystallization (TRAC) method as shown in Figure 1 (see details in the Supporting Information). First, a PDMS template is prepared (Figure 1a). Different from the reported template methods,^31,32^ to facilitate the growth of CsCu_2_I_3_ microwires, the two ends of the prepared PDMS template were removed to expose the microchannels (Figure 1b). When the precursor is dripped to one end of the multiple channels, the solution will go into the channel automatically (Figure 1c) and crystallize (Figures 1d and S1j and Movie S1 optical microscope diagram). Guided by capillary force, the precursor forms a square-shaped capillary trailing,^31,33,34^ resulting in the precursor being supersaturated at the trailing end. In addition, PDMS templates restrict the crystallization position of microwires, enabling the formation of controllable microwire arrays on the substrate (Figure 1f). The excellent controllability of the template method in the preparation of micro- and nanodevices is well illustrated, which encompasses positional adaptability of the prepared materials, with spacing ranging from 10 to 80 μm (Figure S1a–d), and the inclusivity of various substrates, such as glass and Si substrates (Figure 1g). Simultaneously, the microwires are uniformly arranged, and large area arrays can be easily obtained (exceeding 6 × 3 mm^2^ as shown in Figure S2). Meanwhile, each microwire shows a rectangular cuboid structure with a smooth surface and no cracks (Figure 1h). EDS analysis (Figure 1i–k) reveals a homogeneous element distribution of Cs (green), Cu (blue), and I (red) across the microwire crystallization region. The atomic ratio of 1:2:3 (Figure S1k) aligns well with the stoichiometry of CsCu_2_I_3_.

**Figure 2:**
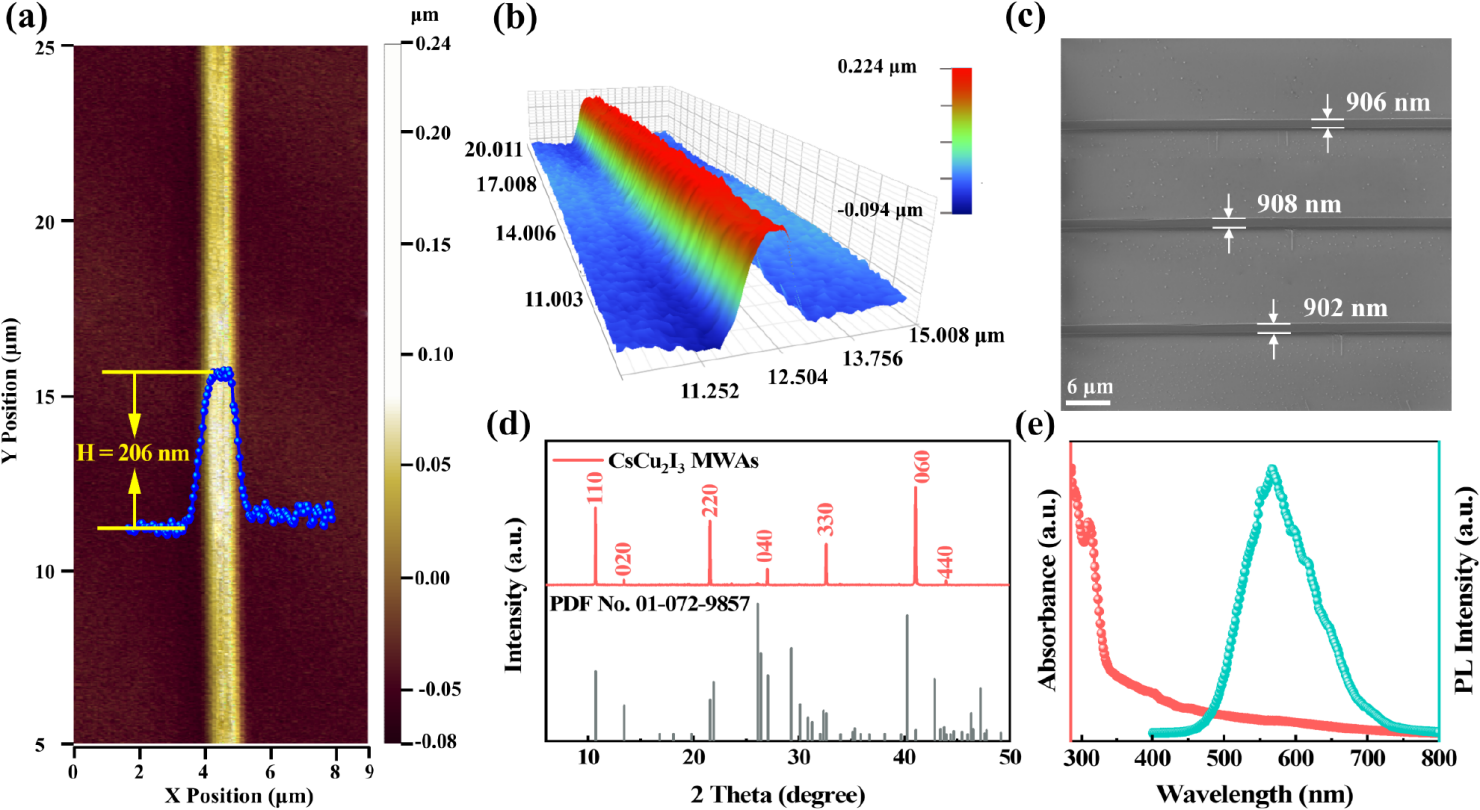
Morphology and optical characterizations of CsCu_2_I_3_ MWAs. 3D optical profilometer (a) plane image and (b) 3D image of a single CsCu_2_I_3_ microwire; (c) SEM image in SE2 mode. XRD pattern (d), absorption, and PL spectrum (e) of CsCu_2_I_3_ MWAs on the glass substrate.

The morphology and spatial characteristics of the CsCu_2_I_3_ MWAs are further confirmed using the three-dimensional optical profiler (Figure 2a,b). The height of the CsCu_2_I_3_ microwire is approximately 206 nm, and the 3D contour illustrates that the microwire owns a uniform height along the entire length. Additionally, the average width of the CsCu_2_I_3_ MWs is determined to be 905 nm by SEM (Figure 2c). While the TRAC method achieves good control of the morphology, achieving excellent crystalline quality is also crucial for a high-performance detector. According to the XRD pattern shown in Figure 2d, the crystal structure of fabricated CsCu_2_I_3_ MWAs can be indexed to the orthorhombic CsCu_2_I_3_ structure in space group *Cmcm* (PDF No. 01-072-9857).^22,27^ The results demonstrate that CsCu_2_I_3_ MWAs have high phase purity and preferred crystalline orientation with either the (110) or (010) planes facing upward. The (110) diffraction peak exhibits a narrow fwhm of 0.043° (Figure S3), signifying the excellent crystalline quality of our synthesized samples. Furthermore, the absorption spectrum (Figure 2e red line) exhibits a sharp edge at 335.4 nm, which corresponds to the characteristic edge of the CsCu_2_I_3_ perovskite with an optical bandgap of 3.70 eV.^35^ When excited by 325 nm wavelength, the PL spectrum (Figure 2e) peak is located at 566 nm, which can be attributed to the typical self-trapped exciton (STE) emission of CsCu_2_I_3_ materials.^36,37^

**Figure 3:**
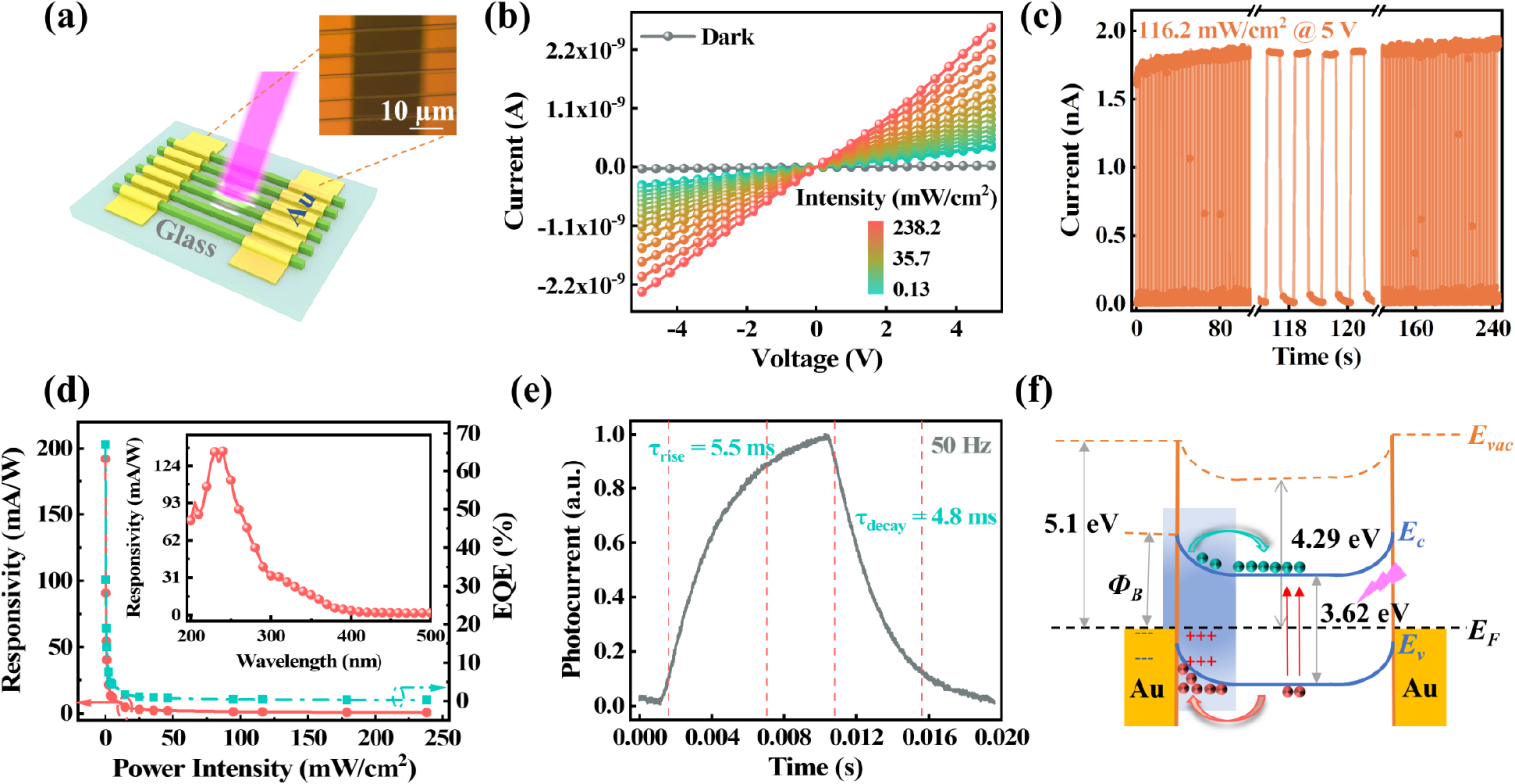
Photoelectric performances of the symmetrical CsCu_2_I_3_ MWA UV photodetector. (a) Schematic and optical microscope image of the fabricated symmetrical CsCu_2_I_3_ MWA device for photoelectrical measurement. (b) Dark current and photocurrent of the device under 355 nm laser illumination at different power densities. (c) Periodic photocurrent response of the photodetector with the power intensity of 116.2 mW/cm^2^ under a frequency of 1 Hz with a 5 V bias. (d) Power-dependent responsivity and external quantum efficiency (EQE) of the photodetector under a 5 V bias. The inset shows the response spectrum of the device. (e) Rising and falling edges of the photodetector for estimating the rise time (τ_rise_) and fall time (τ_decay_) at 5 V bias and 50 Hz. (Estimation methods: the rise time (τ_rise_) from 10% to 90% and the decay time (τ_decay_) from 90% to 10% of its peak value.^44^) (f) Energy band diagram of the device under 355 nm laser illumination at zero bias.

The excellent crystal quality and optical characteristics of the CsCu_2_I_3_ MWAs lead to good performance in detecting UV light. A simple Au/CsCu_2_I_3_/Au symmetric MSM structure detector is fabricated (Figure 3a). The microscopy photograph (Figure 3a inset) depicts the device with a channel width of 20 μm, length of 1 mm, and microwire spacing of approximately 8 μm. Figure 3b displays the *I*–*V* curves of the symmetric device under dark conditions and with 355 nm laser illumination. The photocurrent of the device is highly reliant on the light power intensity and increases significantly from 29.6 pA to 2.7 nA with the optical power density increasing from 0 to 283.2 mW/cm^2^ at 5 V bias. This indicates that CsCu_2_I_3_ efficiently absorbs UV light and converts it into photocurrent under a bias, achieving a maximum light switch ratio of 90 (Figure S5a). In addition, the symmetric linear *I–V* curve implies a good metal–semiconductor contact interface. Figure 3c shows the time-resolved optical response characteristics of the device. Under high-intensity (116.2 mW/cm^2^) UV light cyclic irradiation (1 Hz optical frequency), the device exhibits high stability and good repeatability for over 250 s in air.

The responsivity (*R*), specific detectivity (D*), and external quantum efficiency (EQE) of the Au/CsCu_2_I_3_/Au device to 355 nm radiation are calculated using the equation (SQ1–SQ3). The symmetric device attains the highest *R*, EQE, and D* of 192 mA/W, 67%, and 2.32 × 10^11^ Jones, respectively, at a maximum driving voltage (5 V) and minimum light power intensity (0.13 mW/cm^2^) (Figures 3d and S5d). Furthermore, the responsivity of the device is linearly voltage-dependent (Figure S5b), indicating that the bias is the only impetus. The power-dependent photocurrent (Figure S5c) is fitted by the power function (). The fitting index β of 0.334 is much smaller than 1, indicating a certain degree of carrier recombination even under 5 V bias, which may result from the strong recombination of exciton pairs in the transmission process attributed to soft lattice-induced ‘excited-state defects’ in CsCu_2_I_3_.^38^ Even so, the symmetric device achieves a rise time of 5.9 ms and a decay time of 4.8 ms (Figure 3e), comparable to the best Pb^2+^-based perovskite UV photodetectors.^39^ The response spectrum of the symmetric CsCu_2_I_3_ device (Figure 3d inset) exhibits a significant response in the solar-blind UV region (200 nm–280 nm), with peak responsivity at 240 nm, which is about ten times higher than that at 355 nm. This characteristic indicates that CsCu_2_I_3_ has great potential in solar-blind UV detection.

**Figure 4:**
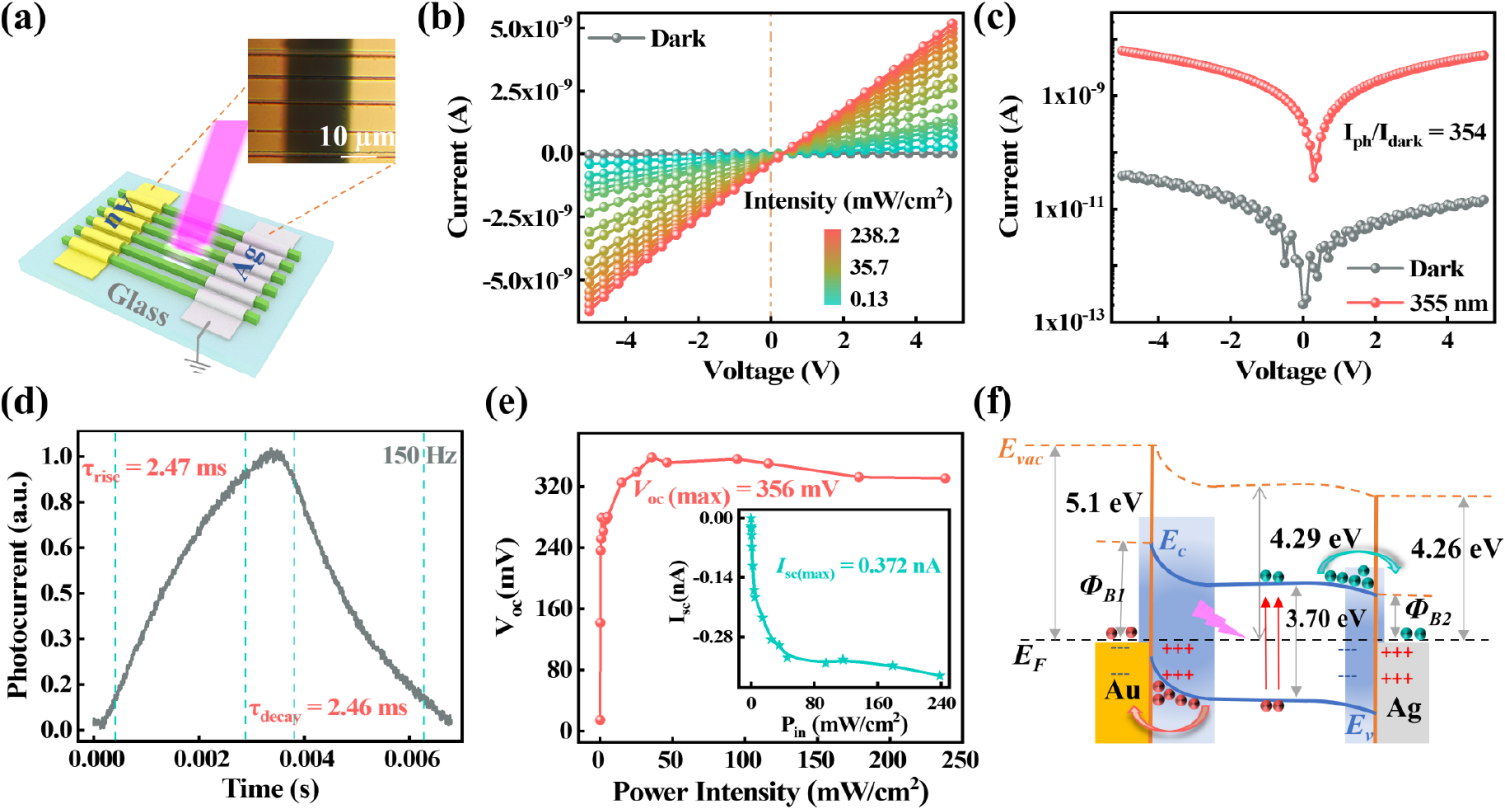
Photoelectric performances of the asymmetric CsCu_2_I_3_ MWA UV photodetector. (a) Schematic and optical microscope image depicting the fabrication of the asymmetric CsCu_2_I_3_ MWA device on the glass substrate. (b) *I*–*V* curves of the device under 355 nm laser illumination with different power densities at −5 to 5 V bias. (c) *I*–*V* curves of the photodetector under dark (black line) and 355 nm laser illumination (red line). (d) Temporal photoresponse of the asymmetric device under 355 nm laser with a frequency of 150 Hz at −5 V bias. (e) Open-circuit voltage (*V*_oc_) versus light intensity of the device. Inset is the short-circuit current (*I*_sc_) curve. (f) Energy band diagram of the asymmetric photodetector under 355 nm laser illumination at zero bias.

To improve carrier separation, an asymmetric device was constructed with Ag and Au as the electrodes (Figure 4a). The device shares physical parameters identical to those of the symmetric one (detailed in the Supporting Information). Under various illuminations, the device current and voltage also exhibit a linear correlation (Figure 4b), implying a good contact interface. This contrasts with the nonlinear *I*–*V* curve caused by Fermi-level pinning that typically occurs at the metal–semiconductor contact interface in most semiconductors.^40^ It is noteworthy that the circuit current of the device exhibits obvious asymmetric and photovoltaic behavior under positive bias and reverse bias. Under the power density of 238.2 mW/cm^2^, the photocurrents at 5 V and −5 V bias exhibit a 21% difference. The light switch ratio (*I*_ph_ /*I*_dark_) reaches 354, three times higher than that of the symmetric device (Figure 4c). Under the bias voltage of −5 V and light power intensity of 0.13 mW/cm^2^, *R*, D*, and EQE are calculated to be 233 mA/W, 3.4 × 10^11^ Jones, and 82%, respectively, as shown in Figure S6a and b. The performance of asymmetric devices is better than most reported perovskite UV photodetectors^39^ (Table S1). Meanwhile, the response times of the asymmetric device (Figure 4d) are determined to be as fast as 2.47 ms (tise time τ_rise_) and 2.46 ms (decay time τ_decay_). Obviously, compared with symmetrical devices, the additional built-in potential accelerates the transmission process. The detection capability of the asymmetric device for UV light is reflected by the different optical switching frequencies (Figure S6d). The device has a response bandwidth of 432 Hz, indicating that its fastest effective optical response speed can be achieved in less than a millisecond.

The obvious photovoltaic behavior under different optical power densities can be observed at a bias of 0 V (Figure S6c). The short-circuit current (*I*_sc_) and open-circuit voltage (*V*_oc_) are extracted in Figure 4e. Due to the contribution of the dark current of the Ag–CsCu_2_I_3_ junction, the *V*_oc_ is small at a low power intensity. With an increasing optical power, it stabilizes at ∼356 mV. The short-circuit current changes synchronously with the incident power intensity, reaching a maximum of 372 pA (238.2 mW/cm^2^). Furthermore, the photovoltaic behavior mechanism of the asymmetric MSM structure under UV irradiation can be explained by the metal–semiconductor contact energy band (Figures 3f and 4f). The reported band structure of CsCu_2_I_3_ indicates that the Fermi level is ∼4.29 eV.^41^ The work functions for Au and Ag are about 5.1 and 4.26 eV, respectively. So the Au/CsCu_2_I_3_ contact leads to an electron barrier, while the Ag/CsCu_2_I_3_ contact results in a quasi-ohmic contact. Under UV irradiation, the photoinduced carriers are generated in CsCu_2_I_3_ microwires, and the electrons and holes are driven and separated by the external bias and built-in electric field due to the metal–semiconductor contacts. In the symmetric device (Figure 3f), when the device receives external light at 0 V bias, electrons are transmitted inside the microwires, and holes reach the contacts. This forms a current from the inside to the surface of microwires in the device. Simultaneously, the movement of the carriers creates a concentration gradient, leading to a current in the opposite direction. Without external bias voltage, the currents in the microwires offset each other. In the asymmetric device (Figure 4f), by matching Au and Ag as electrode materials, the built-in electric field is dominated by the Au/CsCu_2_I_3_ junction with a wide depletion region. Even under 0 V bias, due to the built-in electric field, the photoinduced electrons can be driven to the Ag electrode side, and the holes can be attracted to the Au electrode side. Consequently, the current from Au to Ag moves within the device circuit. In the device test, the Au electrode serves as the voltage-added end, and the Ag electrode acts as the grounding end. Therefore, the positive *V*_oc_ and negative *I*_sc_ observed in Figure 4e are generated. The current in the circuits of both symmetric and asymmetric devices can be effectively modeled by the thermoelectric emission model, with detailed deductions and analyses provided in the Supporting Information.

**Figure 5:**
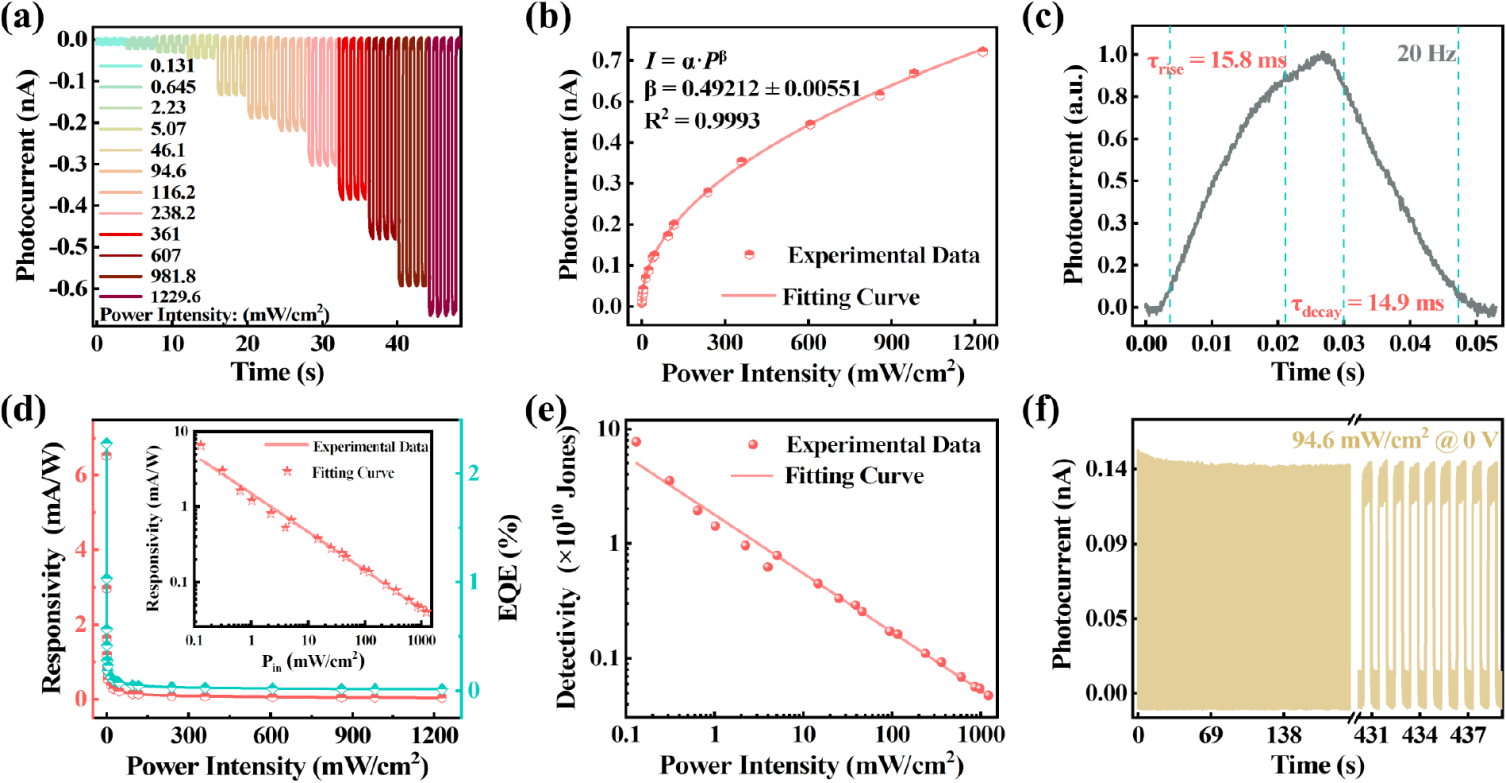
Self-driven performances of the asymmetrical CsCu_2_I_3_ MWA UV photodetector. (a) *I*–*T* curves of the device operated with light power intensity ranging from 0.131 mW/cm^2^ to 1229.6 mW/cm^2^ at 0 V bias. (b) Photocurrent as a function of light density and the corresponding current-power fitting curve at 0 V bias. (c) Typical rise and decay times of the device calculated from one response cycle under 0 V bias at 20 Hz. Power-dependent (d) responsivity(black line), external quantum efficiency (EQE) (red line), and (e) detectivity of the self-driven device. The inset shows the logarithmic responsivity–power curves and line fitting curve of the self-driven device. (f) Periodic photocurrent response of the photodetector under 94.6 mW/cm^2^ power intensity at 1 Hz.

To further verify the self-driven behavior of the CsCu_2_I_3_ MWA UV photodetector, photoelectric tests were conducted under the 0 V bias. At different incident light intensities, the self-driven current demonstrated a significant and rapid increment, reaching a maximum photocurrent of 684 pA at 1229.6 mW/cm^2^ (Figure 5a). Compared with dark current, the on/off ratio (*I*_ph_/*I*_dark_) of the device is proportional to the light power density, with the maximum *I*_ph_*I*_dark_ reaching 2 × 10^3^ (Figure S8a). Meanwhile, the LDR of the UV photodetector further increased from 51.3 dB (Figure S4) to 71.9 dB (Figure S7). Figure 5b shows the relationship between the self-driven current and the power density of the UV light. The β factor is 0.49 by exponential fitting (), which is higher than the value of the all of above devices. This indicates that the device in the self-driven mode has stronger carrier separation, providing ample sensitivity to respond to fluctuations in weak optical signals. Additionally, the performance of the device operating in photovoltaic mode is further analyzed. The response speed of the self-powered device was determined with a τ_rise_ and τ_decay_ of 15.8 and 14.99 ms, respectively (Figure 5c). Under a power density of 0.131 mW/cm^2^, the *R* and EQE of the self-powered device are 6.5 mA/W and 2.3%, respectively (Figure 5d). In the self-drive mode, the dark current in the device is negligible, enabling the device to achieve the same detectivity level as when driven by an external bias of 5 V (Figure 5e). This implies that by introducing simple asymmetric electrodes, the power consumption of the device can be minimized while maintaining its functionality. The stability of the self-driven device is also evaluated, which is particularly important when the device is operated independently. The photocurrent has ultralow fluctuations over 440 s of light on/off switching cycles (Figure 5f). Subsequently, the device is stored in the air for 3 months, followed by tens of thousands of light cycle tests under the same conditions (Figure S9). Remarkably, the self-driven device demonstrated almost no attenuation in its UV response.

## Conclusions

In conclusion, we have successfully constructed a high-performance self-driven UV photodetector on high-quality CsCu_2_I_3_ MWAs utilizing an asymmetric metal contact design. The response speed of the asymmetric device with Au–Ag electrodes was determined, with respective times of 2.47 and 2.46 ms. In addition, the device showed obvious photovoltaic characteristics, featuring a *V*_oc_ of 356 mV. When working in self-driven conditions, the device showed a high UV on/off current ratio up to 10^3^, and the detectivity remained consistent with that under applied bias. Moreover, self-driven behavior enhances the stability of the device, enabling it to operate steadily over tens of thousands of optical switching cycles, with virtually no current decay compared to its initial state even after three months of exposure to air. Our demonstration provides a practical pathway toward establishing stable perovskite UV detectors and lays the foundation for the practical application of low-cost, environmentally friendly, on-chip independently operating UV detectors.

### Experimental Section

#### Synthesis of the Precursors

The raw materials 5.196 mg of CsI (Xìan Polymer Light Technology Co., Ltd., 99.99%) and 7.618 mg of CuI (MACKLIN Reagent Co., Ltd., 99.99%) were dissolved in a solvent mixture of 200 mL of dimethyl sulfoxide (Aladdin Chemistry Co., Ltd., DMSO, 99.9%) and 800 mL of *N*,*N*-dimethylformamide (Aladdin Chemistry Co., Ltd., DMF, 99.9%). The mixture was constantly stirred at room temperature until a clear pale-yellow solution was obtained.

#### Si Template Fabrication

First, the silicon template was prepared by photolithography and ICP etching.^42^ The specific methods were as follows: a photoresist AZ MIR 701 (MicroChemicals, Germany) was spin-coated at 600 rpm for 90 s on a 4 in. bare Si wafer and baked on a hot plate at 100 °C for 120 s. With the exposure energy of 80 mJ cm^–2^, the designed pattern was exposed on the silicon wafer, and the postexposure baking was set to 120 °C for 120 s. Development was conducted using a Microposit MF 319 instrument for 60 s. Subsequently, the pattern was transferred into the Si wafer using the mask during ICP etching with a SF_6_ and O_2_ gas flow ratio of 87/43 sccm, source radio frequency (RF) power of 600 W, bias RF power of 30 W, and gas pressure of 35 mTorr. The height of the pillar pattern was controlled by carefully adjusting the dry etching time. The photoresist mask was removed in a stripper solution (SYS 9072, Sinyang, China). Then, the Si template was cleaned via RAC cleaning steps and dried it. Hydrophobic treatment of the Si template was needed to remove PDMS from the Si template.^43^ The deposition of self-assembled monolayer (SAM) of 1*H*,1*H*,2*H*,2*H*-perfluoro-decyl trichlorosilane (FDTS, Adamas-beta) was done as follows: an excess of FDTS (1 μL) was syringed on a glass bottle adjacent to 25 × 25 mm^2^ Si template, while the temperature was maintained at 120 °C using a hot plate. Afterward, vacuum pumping at a volume of 3 L and a chamber pressure of ∼0.15 atm for 30 min completes the thermal evaporation of the hydrophobic layer.

#### Fabrication of the PDMS Template

The silicon elastomer (SYLGARD-184) and curing agent were blended in a mass ratio of 10:1. After vacuum pumping at a chamber pressure of −0.1 MPa for 10 min in a vacuum oven, the mixture was spin-coated on a hydrophobic silicon template at 300 rpm for 30 s. Then, it was placed on a hot table at 120 °C for 5 min to solidify the PDMS completely. After the mixture was cooled, the graphical PDMS templates were easily torn off.

#### Growth of CsCu_2_I_3_ MWAs

The PDMA template was cut to expose both ends of the channel, transferring it to a clean glass substrate. Note: here, the glass substrate did not require UV-ozone treatment, and the template could be pressed to ensure PDMS contact with the substrate completely. Followed by, a small amount of CsCu_2_I_3_ precursor (3 μL) was dropped at one end of the template, and the precursor filled the channel through capillary action. Heating at a low temperature (60 °C), the precursor inside the channel crystallized slower than at the edge of the channel. It moved from the dripping end to the other end with a concave meniscus, crystallizing on both sides of the channel sidewall to form microwires. After 1 h, the solvent was completely volatilized, the PDMS template was gently peeled off, and uniform microwire arrays (MWAs) were fabricated on substrates.

#### Fabrication of the CsCu_2_I_3_ MWA Photodetector

(1) Using the physical mask method, a 25 μm gold wire was fixed on the MWAs, and then, the electrode with a width of 1 mm was fabricated by thermal evaporation of gold. After completion, the gold wire was removed to form a device with a channel of about 20 μm. (2) Asymmetric devices were fabricated using a two-step shadow mask. Similarly, the microwires were covered with gold wires, and then, a half-patterned mask plate was used to thermally vaporize gold and silver in two steps to form different electrodes with a channel of about 20 μm and a width of 1 mm.

Note: a simple physical gold-wire mask was used to fabricate the device channel, to avoid defects inducing on the smooth surface of the MWAs and causing unfavored electrical contact. The channel width will be slightly narrowed due to the diffusion of hot vapor metal ions during evaporation.

#### Characterization of the CsCu_2_I_3_ MWAs

The morphologies of the microwires were obtained by scanning electron microscopy (SEM, Sigma FE-SEM, Zeiss, Germany). Before observation, all substrates were sputter-coated with a thin layer of gold to enhance conductivity. Energy-dispersive X-ray spectroscopy (EDS) mapping was acquired by a Zeiss FE-SEM instrument. Optical Profiler (ContourGK-K, Bruker Nano, Inc., US) was used to study the three-dimensional images of MWAs and Si templates to measure their height and width under white light (VSI mode) at 100 nm (microwires) and 50 μm (template) backscan. The crystal structure of the CsCu_2_I_3_ MWAs was characterized by X-ray diffraction (XRD; Smartlab, Rigaku, Japan). The XRD system uses a rotating anode X-ray source with Cu (λ ∼ 1.54), and the step was 0.01 degree. The PL spectra were studied by using a Confocal Laser Raman Spectrometer (LabRAM HR Evolution, HORIBA, Japan) with a 325 nm excitation laser. The optical images of all microwires were captured with a Zeiss AxioCam ICC 5 microscope.

The electrical characteristics (*I*–*V*, *I*–*T*) were measured on a semiconductor device analyzer (B1500A, Keysight, US) equipped with a probe station (Semishare, China) and a silver probe. The excitation light source of the device adopted a frequency-doubled laser (Genesis CX SLM series, COHERENT, US) at 355 nm with a maximum power intensity of 38.6 mW and a facular area of 3.14 × 10^–2^ cm^2^. The response speed was measured by using a mixed domain oscilloscope (MDO4054C, Tektronix, US) in conjunction with a low-noise current preamplifier (SR570, Stanford Research Systems, US), and the optical switch of the laser source was controlled with a function/arbitrary waveform generator (DG4602, RIGOL, China). The response spectrum under different wavelengths was measured using a response measurement system equipped with a monochromator (Zolix Instruments, China), a lock-in amplifier (SR830, Stanford Research Systems, US), and a 150 W xenon lamp.

## Supporting Information Available

Experimental details include morphology and structure of the CsCu_2_I_3_ MWAs; optical microscope image of the large-area CsCu_2_I_3_ MWAs; the analysis of the basic performance for a photodetector; performances of the symmetrical CsCu_2_I_3_ MWA UV photodetector; device performances of the asymmetric CsCu_2_I_3_ MWA UV photodetector; performance of UV detectors with different active materials; the theoretical analyze based on thermal electon emission model; performance analysis of the self-driven CsCu_2_I_3_ MWA UV photodetector; environmental stability of the self-driven device (PDF)
A movie documenting the process of microwire preparation (MP4)
