# Supplementary material for: Air-Stable Self-Driven UV Photodetectors on Controllable Lead-Free CsCu2I3 Microwire Arrays
Source: ACS Appl Mater Interfaces. 2024 Feb 21;16(8):10398–406. doi: 10.1021/acsami.3c17881 (PMC10910456; doi:10.1021/acsami.3c17881)
Supplement: Supplementary file 1 — am3c17881_si_001.pdf [file am3c17881_si_001.pdf]

Supporting Information

Air-Stable Self-Driven UV Photodetectors on Controllable Lead-Free $CsCu_2I_3$ Microwire Arrays

Zhi-Hong Zhang, [#,1†,2‡] Shan-Shan Yan,[#,2‡] Yu-Long Chen,[2‡] Zhen-Dong Lian,[2‡] Ai Fu,[2‡] You-Chao Kong,[2‡] Lin Li,[3§] Shi-Chen Su,[4||] Kar-Wei Ng,[*,2‡] Zhi-Peng Wei,[*, 1†] Hong-Chao Liu,[*,2‡] and Shuang-Peng Wang[*,2‡]

[†] State Key Laboratory of High Power Semiconductor Lasers, Changchun University of Science and Technology, Changchun 130022, China.

[‡] Institute of Applied Physics and Materials Engineering, University of Macau, Macao, Taipa, Macao SAR 999078, China

[§] Key Laboratory for Photonic and Electronic Bandgap Materials, Ministry of Education, School of Physics and Electronic Engineering, Harbin Normal University, Harbin 150025, China

[||] School of Semiconductor Science and Technology, South China Normal University, Foshan 528000, China

[#]These authors contributed equally.

* Email: billyng@um.edu.mo

* Email: zpweicust@126.com

* Email: hcliu@um.edu.mo

* Email: spwang@um.edu.mo. Tel: +853 8822 4048.

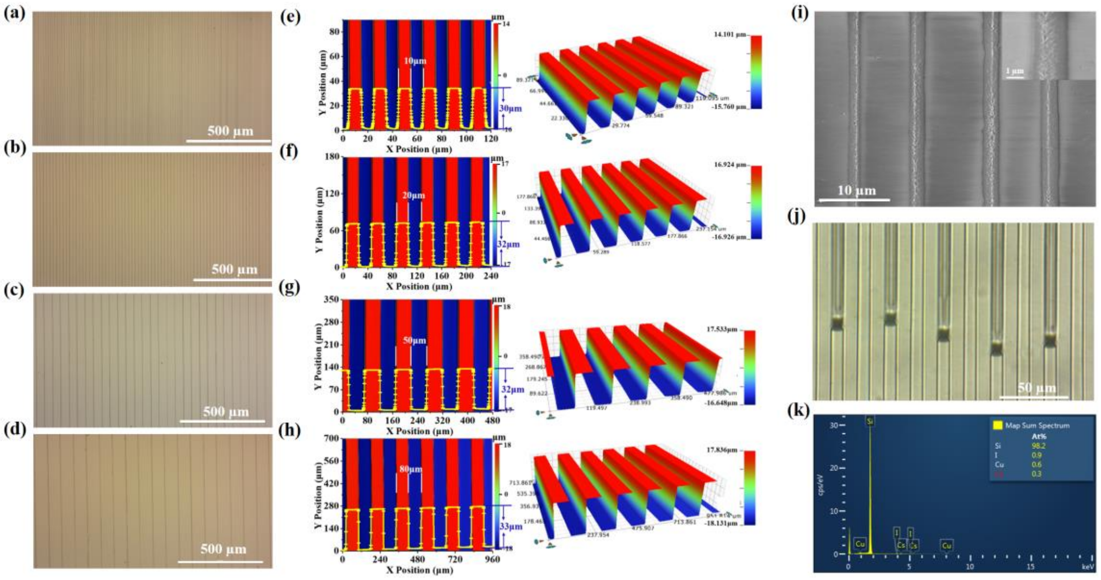

Figure S1. Morphology and structure of the $CsCu_2I_3$ MWAs. (a–d) Optical microscope image of different spacing of large area $CsCu_2I_3$ MWAs on the glass substrate. (a) 10 μm; (b) 20 μm; (c) 50 μm; (d) 80 μm. (e–f) 3D optical profilometer image of silicon template with different distances. Left: plane images; Right: 3D images. Inset: the width and depth data of Si templates. (i) SEM images of $CsCu_2I_3$ MWAs with the precursor of natural crystallization. (j) Optical microscope images of microwires crystallization proceeding in PDMS template. (k) EDS spectrum of the $CsCu_2I_3$ MWAs.

Through photolithography and inductively coupled plasma (ICP) etching[1], the Si template (25 × 25 mm^2) with uniform depth (about 30 μm) and regular sidewall is fabricated. As shown in Figure S1 e–h, the templates with different widths (10 μm, 20 μm, 50 μm, and 80 μm) are etched respectively. The three-dimensional optical profiler on the right side fully shows the three-dimensional morphology of the prepared Si template. Figure 1a shows the divestiture process of nanoimprinted lithography (NLP) PDMS (15 × 15 × 5 mm^3) on the Si template. To make PDMS easy to be stripped from the Si template, the self-assembled monolayer (SAM) films (1H, 1H, 2H, 2H-perfluoro-decyl trichlorosilane, FDTS) are performed on the Si template ahead of schedule, which also led some hydrophobic molecules to the PDMS template under heating conditions[2]. When the PDMS template is transferred to the glass substrate, it is

necessary to cut the two ends of the template to expose the channel, which guarantees the precursor solution can fill the whole template channel and evaporation in the microchannel, as shown in Figure 1b and 1c. Figure 1d and Figure S1j illustrate the crystallization process of the precursor. Under the guidance of capillary force, the precursor in the middle of the channel is gradually replenished to the tail, leaving the precursor supersaturation at that point[3, 4]. In addition, the glass substrate without any treatment and the PDMS sidewall adhered to FTDS molecules limit the crystallization of the fan-shaped capillary trailing to the gap between the two sides. Thus, microwires with uniform straight shapes and continuous crystallization are formed as shown in Figure 1e. It is worth noting that the crystallization of the precursor must be carried out under heated conditions. Under the crystallization mode of natural evaporation, the slow crystallization mode will widen the capillary-trailing edge and make the crystallization discontinuous, resulting in the surface collapse and fish-scale cracks of microwires as shown in Figure S1i. On the other hand, the PDMS can be easily peeled off after complete crystallization, which will not breakage microwires (Figure 1e–f). In addition, the TRAC method is also very flexible to synthesize MWAs with different requirements. The distribution of MWAs on the substrate can be changed by adjusting the template style according to the requirement. For example, Figure S1a–d shows the MWAs with different spacings (10 μm, 20 μm, 50 μm, and 80 μm). The width and height of the microwire can be adjusted by adjusting the concentration of the precursor[5, 6].

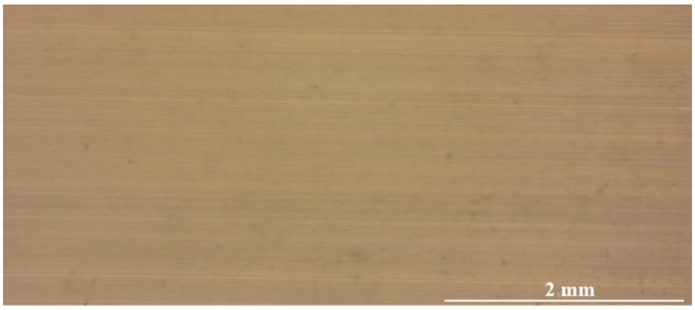

Figure S2. Optical microscope image of the large-area $CsCu_2I_3$ MWAs.

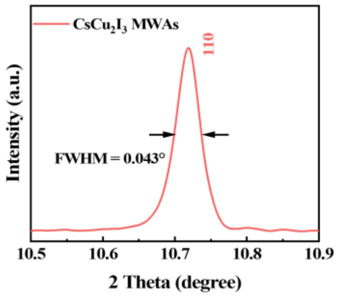

Figure S3. The enlargement of (110) lattice phase XRD diffraction peak of $CsCu_2I_3$ MWAs.

The most basic performance metric for a photodetector is responsivity (R), specific detectivity (D*), and external quantum efficiency (EQE) can be calculated from the following equation:

$$R = \frac{I_{ph} - I_{dark}}{P_{\mathrm{light}} \cdot S} \quad \text{(SQ1)}$$

$$D^* = \frac{S^{\frac{1}{2}} \cdot R}{(2 \cdot e \cdot I_{\mathrm{d}})^{\frac{1}{2}}} \quad \text{(SQ2)}$$

$$EQE = \frac{R \cdot h \cdot c}{e \cdot \lambda} \quad \text{(SQ3)}$$

$$LDR = 20 \, log \, (\frac{P_{max}}{P_{min}}) \quad \text{(SQ4)}$$

where I_{ph} and I_{dark} are the photocurrent and dark current, e is the elementary charge, h

is the Planck constant, c is light speed, and λ is the light wavelength, respectively. P_{light} is the incident light power intensity which can be expressed as $P_{light} = p \cdot s$, where p is the output power of 355 nm laser, and s is the light spot area. S is the effective area of the device, which can be denoted as $S = n \cdot l \cdot w$, where n is the number of microwires in the channel, l is the channel length, and w is the width of microwires. P_{max} and P_{min} are the power densities of maximum and minimum irradiated light for a linear response current.

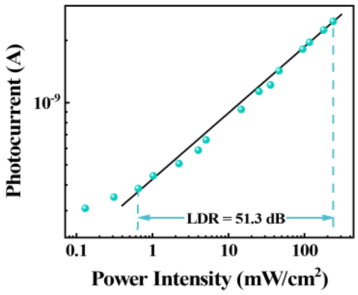

Figure S4. Photocurrent versus incident power plot of the symmetrical $CsCu_2I_3$ MWA UV photodetector at 5 V bias.

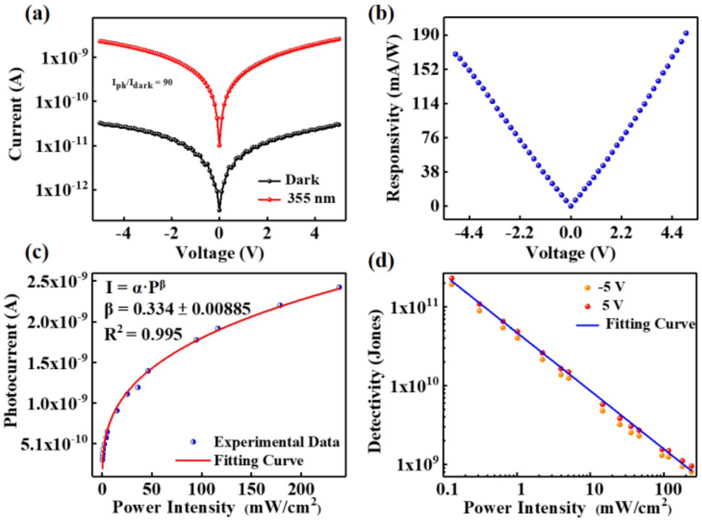

Figure S5. Performances of the symmetrical $CsCu_2I_3$ MWA UV photodetector. (a) *I-V* curves of the UV photodetector under dark (black line) and 355 nm laser illumination (red line). (b) Responsivity of the photodetector at different biases under 0.13 mW/cm^2 power intensity. (c) The photocurrent as a function of light density and the corresponding current-power fitting curve at 5 V bias. (d) Power-dependent versus

detectivity and line fitting curve (blue line) of the device under 5 V (red point) and −5 V (orange point) bias.

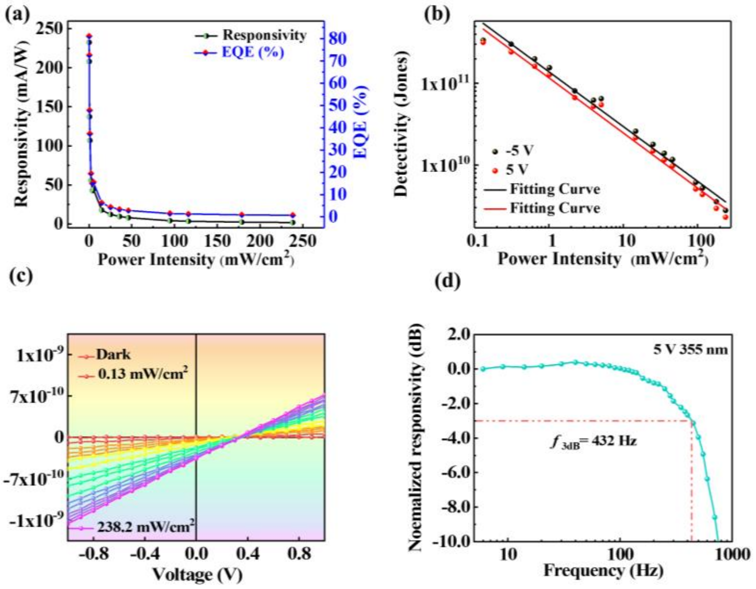

Figure S6. Device performances of the asymmetric $CsCu_2I_3$ MWA UV photodetector. (a) Power-dependent responsivity (black line) and external quantum efficiency (EQE) (blue line) of the device at 5 V bias (b) Power-dependent detectivity and line fitting curve (blue line) of the device at 5 V (red point) and −5 V (black point) bias with the light power density ranging from 0.13 mW/cm^2 to 238.2 mW/cm^2. (c) The amplified *I–V* curve of asymmetric devices under ± 1 V bias. (d) Frequency response of the device, showing the -3dB cutoff frequency of 432 Hz.

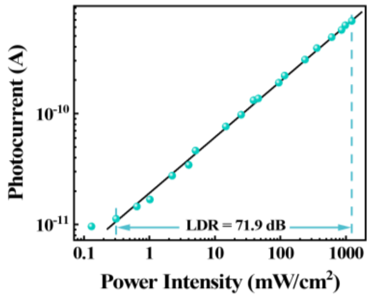

Figure S7. Photocurrent versus incident power plot of the asymmetrical $CsCu_2I_3$ MWA UV photodetector at 0 V bias.

Table S1. Performance of UV detectors with different active materials.

Device type	Bias (V)	Responsivity (mA/W)	Time (ms)	LDR (dB)	Stability	Ref
Ag/NiO/Ga_2O_3/Ag	0 V	5.7×10^{-2} @ (245 nm)	340/3600	-	-	[7]
In/Ag/GaN/Ga_2O_3	0 V	28.44 @ (254 nm)	140/70	-	-	[8]
In/ZnO homojunction nanofibers	0 V	1 @ (solar-like UVA)	3.9/4.7 ($\times10^3$)	-	-	[9]
QDs/ITO/$MAPbI_3$/ PCBM/BCP/Ag	-0.1 V	1.4 @ (279 nm)	< 70	83.7	-	[10]
Au interdigital/$CsPbCl_3$	5 V	14.3 @ (325 nm)	3.212/2.511	-	9 h (373 K)	[11]
ITO/$CsPbCl_3$/ITO	4 V	2110 @ (365 nm)	77/63	57	2000 s	[12]
Au/$CsPbCl_3$	0.5 V	3.64×10^3 @ (265 nm)	1.84/3.3	107/ LDR=20log(I_p/I_d)	30 day	[13]
Au/$MAPbCl_3$/Pt	15 V	46.9 @ (365 nm)	24/62	-	0.5 day	[14]
Au interdigital/$CsCu_2I_3$	5 V	22.1 @ (265 nm)	-/-	-	-	[15]
Ag/$CsCu_2I_3$	10 V	10-52 @ (300-700 nm)	0.188/14.7	-	45 day	[16]
Ni/$CsCu_2I_3$	-5 V	-	50.4/244.8	-	-	[17]
Au/$CsCu_2I_3$ NWs/Au	5 V	89.73 @ 365 nm 12350 @ 310 nm	170.4/290.7	-	30 day	[18]
Au/$CsCu_2I_3$ wire array/Au	5 V	80 @ 365 nm; 1290 @ 330 nm	0.64/7.1	-	-	[19]
Ag/$CsCu_2I_3$/Ag	5 V	192 @ (355 nm)	4.8/5.9	51.3	-	This work
Ag/$CsCu_2I_3$/Au	5 V	233 @ (355 nm)	2.47/2.46	-	-	This work
Ag/$CsCu_2I_3$/Au	0 V	6.5 @ (355 nm)	15.8/14.99	71.9	90 day /11000 cycles	This work

Table related references[7-19].

For perovskite materials, the average free path of carriers is much larger than the barrier width, so the current in the MSM structure can be calculated by the thermal electron emission model.[20, 21]

$$J = J_{Ag} + J_{Au} \tag{SQ4}$$

$$= [J_{0Ag}\left(e^{\frac{qV_{Ag}}{nkT}} - 1\right) + qGW_{Ag}] - [J_{0Au}\left(e^{\frac{-qV_{Au}}{nkT}} - 1\right)$$

$$+ qGW_{Au}]$$

$$J_{0Ag} = A^{*}T^{2}e^{\frac{-q\Phi_{nAg}}{kT}} \tag{SQ5}$$

$$W_{Ag} = \sqrt{\frac{2\varepsilon_{s}(V_{BAg} + V_{Ag})}{qN_{d}}} \tag{SQ6}$$

$$W_{Au} = \sqrt{\frac{2\varepsilon_{s}(V_{BAu} - V_{Au})}{qN_{d}}} \tag{SQ7}$$

Where the total current density (J) of the circuit is divided into the Au-terminal current density (J_{Au}) and the Ag-terminal current density (J_{Ag}). They are composed of two components, namely, the current of the carrier in the device itself under external bias (the first) and the current generated by external optical excitation (the second). $J_{0Ag/0Au}$ is the saturated current density, $V_{Ag/Au}$ is the voltage added to different electrodes, n is the ideal factor, G is the photogenerated carrier rate, $W_{Ag/Au}$ is the depletion layer width of perovskite at the two electrodes, $V_{BAg/BAu}$ is the built-in potential generated by the depletion layer, and s and N_d are the dielectric constant and carrier concentration of $CsCu_2I_3$. And the V_{BAg} can be expressed as $V_{Bag} = \Phi_{nAg} - \chi$, where χ is the electronic

affinity of $CsCu_2I_3$.

According to the above model, when the device is in a short circuit, the first current ($V_{Ag/Au} = 0$) determined by the external bias is zero, the metal and semiconductor are in a dynamic equilibrium, and there is no current in the device circuit. When the external light excitation is added, the device circuit current is generated by the movement of photogenerated carriers, that is, the difference between the two-electrode current of the second item ($qGW_{Au/Ag}$). For symmetric devices, the same contact metal has the same Schottky barrier and the same depletion region width, so the total circuit current is zero. Only when the external bias changes the symmetry of this barrier, there will be current in the circuit. For asymmetric devices, because the work function of Au is higher than that of Ag, they will generate different built-in potentials when they contact with $CsCu_2I_3$, resulting in different width depletion regions, that is, the depletion region of Au is wider than that of Ag. At this point, the difference between the two ends of the current is not zero in the case of a short circuit and external light excitation, thus forming a circuit current. Moreover, when the external voltage is positive (negative), the direction of the external electric field is opposite (same) to that of the built-in electric field, so the asymmetric circuit current is generated.

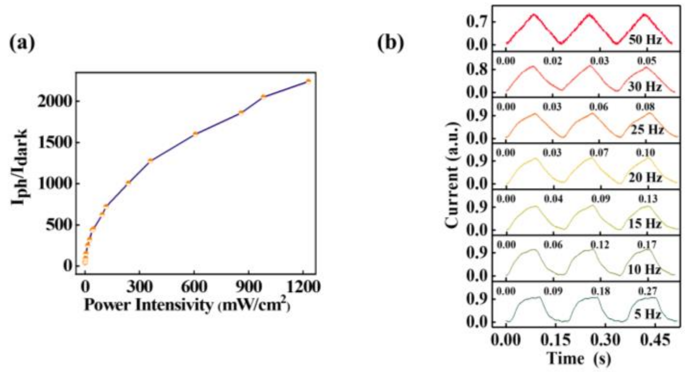

Figure S8. Performances analysis of the self-driven $CsCu_2I_3$ MWA UV photodetector. (a) I_{dark}/I_{light} ratio of the self-driven $CsCu_2I_3$ MWAs UV photodetector as functions of the power density. (b) Time-resolved photoresponse of the self-driven device upon the switching frequency ranging from 5 Hz to 50 Hz (0 V).

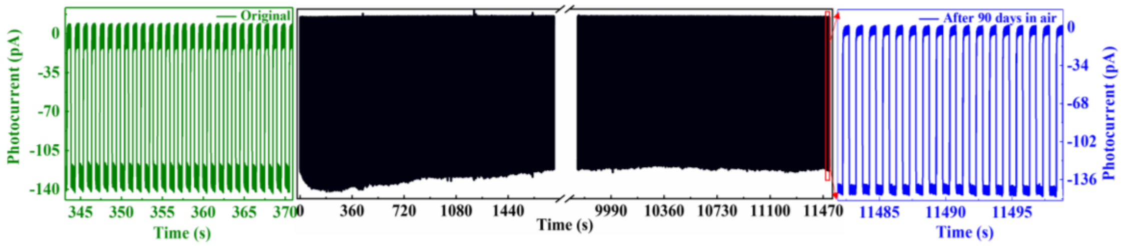

Figure S9. Through *I*–*T* to reflect the environmental stability of the self-driven device. The initial state of the device (400 cycles (green)), after three months (11000 cycles (black)), and after three months of *I*–*T* local amplification (blue).

Reference

1. Li, M.; Chen, Y.; Luo, W.; Cheng, X. Nanoindentation Behavior of UV-Curable Resist and Its Correlation with Patterning Defect in Nanoimprint Lithography. *J. Micromech. Microeng.* **2020**, *30* (6), 065010, DOI: 10.1088/1361-6439/ab87ed
2. Li, M.; Huang, X.; Luo, W.; Chen, Y.; Han, F.; Cheng, X. Thermal Degradation Behavior of Self-Assembled Monolayer Surfactant on Silicon Substrate. *J. Vac. Sci. Techno. B* **2020**, *38* (3), 032602, DOI: 10.1116/1.5143307
3. Feng, J.; Yan, X.; Liu, Y.; Gao, H.; Wu, Y.; Su, B.; Jiang, L. Crystallographically Aligned Perovskite Structures for High-Performance Polarization-Sensitive Photodetectors. *Adv. Mater.* **2017**, *29* (16), DOI: 10.1002/adma.201605993
4. Feng, J.; Gong, C.; Gao, H.; Wen, W.; Gong, Y.; Jiang, X.; Zhang, B.; Wu, Y.; Wu, Y.; Fu, H.; Jiang, L.; Zhang, X. Single-Crystalline Layered Metal-Halide Perovskite Nanowires for Ultrasensitive Photodetectors. *Nat. Electron.* **2018**, *1* (7), 404-410, DOI: 10.1038/s41928-018-0101-5
5. Liu, P.; He, X.; Ren, J.; Liao, Q.; Yao, J.; Fu, H. Organic-Inorganic Hybrid Perovskite Nanowire Laser Arrays. *ACS Nano* **2017**, *11* (6), 5766-5773, DOI: 10.1021/acsnano.7b01351
6. Li, S. X.; Xu, Y. S.; Li, C. L.; Guo, Q.; Wang, G.; Xia, H.; Fang, H. H.; Shen, L.; Sun, H. B. Perovskite Single-Crystal Microwire-Array Photodetectors with Performance Stability Beyond 1 Year. *Adv. Mater.* **2020**, *32* (28), e2001998, DOI: 10.1002/adma.202001998
7. Wang, Y.; Wu, C.; Guo, D.; Li, P.; Wang, S.; Liu, A.; Li, C.; Wu, F.; Tang, W. All-Oxide NiO/Ga_2O_3 P–N Junction for Self-Powered UV Photodetector. *ACS Appl. Electron. Mater.* **2020**, *2* (7), 2032-2038, DOI: 10.1021/acsaelm.0c00301
8. Li, P.; Shi, H.; Chen, K.; Guo, D.; Cui, W.; Zhi, Y.; Wang, S.; Wu, Z.; Chen, Z.; Tang, W. Construction of GaN/Ga_2O_3 P–N Junction for an Extremely High Responsivity Self-Powered UV Photodetector. *J. Mater. Chem. C* **2017**, *5* (40), 10562-10570, DOI: 10.1039/C7TC03746E
9. Ning, Y.; Zhang, Z.; Teng, F.; Fang, X. Novel Transparent and Self-Powered UV Photodetector Based on Crossed ZnO Nanofiber Array Homojunction. *Small* **2018**, *14*

(13), 1703754, DOI: 10.1002/smll.201703754

10. Zou, T.; Liu, X.; Qiu, R.; Wang, Y.; Huang, S.; Liu, C.; Dai, Q.; Zhou, H. Enhanced UV-C Detection of Perovskite Photodetector Arrays Via Inorganic $CsPbBr_3$ Quantum Dot Down-Conversion Layer. *Adv. Opt. Mater.* **2019,** *7* (11), 1801812, DOI: 10.1002/adom.201801812

11. Li, Y.; Shi, Z.; Lei, L.; Ma, Z.; Zhang, F.; Li, S.; Wu, D.; Xu, T.; Li, X.; Shan, C. Controllable Vapor-Phase Growth of Inorganic Perovskite Microwire Networks for High-Efficiency and Temperature-Stable Photodetectors. *ACS Photonics* **2018**, *5* (6), 2524-2532, DOI: 10.1021/acsphotonics.8b00348

12. Zhu, Z.; Deng, W.; Li, W.; Chun, F.; Luo, C.; Xie, M.; Pu, B.; Lin, N.; Gao, B.; Yang, W. Antisolvent-Induced Fastly Grown All-Inorganic Perovskite $CsPbCl_3$ Microcrystal Films for High-Sensitive Uv Photodetectors. *Adv. Mater. Interfaces* **2021,** *8* (6), 2001812, DOI: 10.1002/admi.202001812

13. Hu, H.; Han, Z.; Huang, B.; Dong, Y.; Zou, Y. Intermediate Phase-Assisted Solution Preparation of Two Dimensional $CsPbCl_3$ Perovskite for Efficient Ultraviolet Photodetection. *J. Colloid. Interface Sci.* **2019**, *554*, 619-626, DOI: 10.1016/j.jcis.2019.07.044

14. Maculan, G.; Sheikh, A. D.; Abdelhady, A. L.; Saidaminov, M. I.; Haque, M. A.; Murali, B.; Alarousu, E.; Mohammed, O. F.; Wu, T.; Bakr, O. M. $CH_3NH_3PbCl_3$ Single Crystals: Inverse Temperature Crystallization and Visible-Blind UV-Photodetector. *J. Phys. Chem. Lett.* **2015**, *6* (19), 3781-6, DOI: 10.1021/acs.jpclett.5b01666

15. Yang, J.; Kang, W.; Liu, Z.; Pi, M.; Luo, L. B.; Li, C.; Lin, H.; Luo, Z.; Du, J.; Zhou, M.; Tang, X. High-Performance Deep Ultraviolet Photodetector Based on a One-Dimensional Lead-Free Halide Perovskite $CsCu_2I_3$ Film with High Stability. *J. Phys. Chem. Lett.* **2020**, *11* (16), 6880-6886, DOI: 10.1021/acs.jpclett.0c01832

16. Li, Z.; Li, Z.; Shi, Z.; Fang, X. Facet-Dependent, Fast Response, and Broadband Photodetector Based on Highly Stable All-Inorganic $CsCu_2I_3$ Single Crystal with 1D Electronic Structure. *Adv. Funct. Mater.* **2020**, *30* (28), 2002634, DOI: 10.1002/adfm.202002634

17. Mo, X.; Li, T.; Huang, F.; Li, Z.; Zhou, Y.; Lin, T.; Ouyang, Y.; Tao, X.; Pan, C.

Highly-Efficient All-Inorganic Lead-Free 1D $CsCu_2I_3$ Single Crystal for White-Light Emitting Diodes and UV Photodetection. *Nano Energy* **2021**, *81*, 105570, DOI: 10.1016/j.nanoen.2020.105570

18. An, Y.; Li, S. X.; Feng, J. C.; Xia, H. Highly Responsive, Polarization-Sensitive, Flexible, and Stable Photodetectors Based on Highly Aligned $CsCu_2I_3$ Nanowires. *Adv. Opt. Mater.* **2023**, 2301336, DOI: 10.1002/adom.202301336
19. Xu, X.; Jiang, S.; Fan, C.; Deng, Q.; Shen, L.; Zhang, Q. Centimeter-Scale Growth of Unidirectional $CsCu_2I_3$ Wire Arrays for High Performance UV Photodetectors. *Adv. Opt. Mater.* **2022**, *10* (20), 2201107, DOI: 10.1002/adom.202201107
20. Sze, S. M.; Li, Y.; Ng, K. K., *Physics of Semiconductor Devices*. John Wiley & sons: **2021**.
21. Li, J.-k.; Ge, C.; Jin, K.-j.; Du, J.-y.; Yang, J.-t.; Lu, H.-b.; Yang, G.-z. Self-Driven Visible-Blind Photodetector Based on Ferroelectric Perovskite Oxides. *Appl. Phys. Lett.* **2017**, *110* (14), 142901, DOI: 10.1063/1.4979587
